# Emotional labor, well-being, and professional development among university teachers: a qualitative study from a job demands-resources perspective

**DOI:** 10.3389/fpsyg.2026.1746170

**Published:** 2026-03-12

**Authors:** Haoquan Sun, Huaiyu Chang, Xiaodong Shen

**Affiliations:** 1 School of Education, Northeast Normal University, Changchun, China; 2 School of Fine Arts, Baicheng Normal University, Baicheng, China

**Keywords:** emotional labor, identity work, job demands-resources, professional development, teacher well-being

## Abstract

This qualitative study explores how university teachers experience and manage emotional labor in relation to their professional well-being and development. Ten teachers from a public research-oriented university took part in semi-structured interviews. The data were analyzed using reflexive thematic analysis, guided by the job demands-resources (JD-R) model, emotional labor theory, and research on teacher professional development and identity. Three main themes emerged. First, institutional emotional rules and hidden pressures included expectations to be “always positive” and “always available,” the emotional effects of evaluations and performance metrics, and blurred boundaries between work and personal life. Second, navigating emotional labor described how teachers moved between surface acting, deep acting, and selective authenticity, and how these strategies provided both protection and emotional costs over time. Third, emotional labor as professional learning showed how emotionally challenging experiences became turning points, how understandings of care and responsibility changed across career stages, and how peer support and mentoring supported more sustainable ways of working. Overall, the findings suggest that universities need multi-level responses that reduce structural pressures and create shared spaces where teachers can reflect on and discuss the emotional demands of academic work.

## Introduction

Teacher well-being has become a central concern in contemporary educational research because it is closely tied to teacher effectiveness, classroom processes, and long-term retention in the profession. Integrative work on teachers’ psychological characteristics shows that traits such as self-efficacy, emotional stability, and adaptive motivation are linked to instructional quality, relationships with students and colleagues, and the likelihood of remaining in teaching (Bardach et al., 2021). From a positive psychology perspective, teacher well-being is increasingly conceptualized as a multidimensional construct involving emotional, cognitive, and relational resources that enable teachers to function optimally rather than merely avoid burnout (Sadeghi and Pourbahram, 2024a). Empirical studies further demonstrate that teachers’ psychological well-being is related to work engagement, enjoyment in teaching, and experiences of professional growth, highlighting that well-being is not only an outcome but also a driver of effective teaching and sustainable careers (Wang, 2024; Zhang et al., 2023).

Although much of this literature has focused on school settings, higher education teachers face specific constellations of demands and resources that warrant separate attention. An integrative review of occupational stress and mental health among higher education teachers identifies heavy workloads, precarious contracts, and gendered inequalities as key risk factors for distress and ill-being in academic staff (de Sousa Pinho et al., 2023). Conceptual work on stress and psychological well-being in university environments similarly suggests that academics operate within complex systems of institutional demands, personal resources, and contextual affordances that shape their experiences of strain and coping (de la Fuente and Martínez-Vicente, 2024). Recent quantitative studies confirm that workload, perceived organizational support, and work engagement jointly predict teachers’ psychological well-being, underscoring the relevance of JD-R perspectives in understanding academic staff experiences (Wang, 2024). At the same time, research on university teachers in diverse national contexts shows that corporate social responsibility initiatives, leadership practices, and institutional culture can either buffer or amplify the impact of structural pressures on well-being (Horoub and Zargar, 2022; Wu, 2024).

The changing technological and organizational landscape of higher education further complicates these dynamics. Educational innovations that embed digital technologies into university teacher training and instructional design can transform work practices, role expectations, and professional identities, sometimes in ways that enhance agency and collaboration, but also by introducing new forms of surveillance and workload (Flores-Chacón et al., 2023). Futures-oriented analyses argue that emerging technologies will play an increasingly important role in shaping the conditions of teacher well-being, demanding anticipatory thinking about how digital infrastructures and datafication reshape academic labor (Casebourne and Sitta, 2024). In parallel, studies of teacher well-being across rural, regional, and metropolitan institutions highlight how geographical and structural variations in resources, collegial support, and community connections contribute to distinct well-being profiles and vulnerabilities (Kingsford-Smith et al., 2023). Together, these strands of work suggest that university teachers’ well-being is embedded in multi-layered organizational and technological contexts rather than being solely an individual attribute.

Within these complex settings, emotional processes are increasingly recognized as central to university teachers’ work. Research grounded in emotional labor theory conceptualizes teaching as involving the regulation of felt and displayed emotions through strategies such as surface acting, deep acting, and authentic expression. Drawing on a JD-R framework, multilevel analyses show that leadership styles and organizational climates shape the emotional labor strategies teachers employ and, in turn, their levels of engagement and turnover intention (Huang and Yin, 2024; Harrison et al., 2025). In higher education specifically, recent evidence from Chinese universities indicates that authenticity, defined as the alignment between experienced and expressed emotion, constitutes an effective emotional labor strategy that supports teaching efficacy when sufficient job resources are available (Zheng et al., 2024). These findings point to emotional labor as a key process through which contextual demands and resources are translated into experiences of well-being or ill-being for university teachers.

At the same time, qualitative and interpretive studies add nuance by foregrounding teachers’ own meaning-making around well-being and emotional experience in higher education. Elicited metaphor analyses with teachers in Chinese Universities of the Third Age reveal that well-being is experienced through metaphors of balance, care, and self-realization, embedded in late-life learning communities and social participation (Geng et al., 2024). Studies of academic staff in different institutional settings similarly show that well-being is negotiated through everyday practices of leadership, collegial interaction, and professional learning, rather than being a static psychological state (Horoub and Zargar, 2022; Flores-Chacón et al., 2023). Conceptual models of university stress and well-being emphasize that teachers’ cognitive appraisals and self-regulatory strategies mediate how they respond to institutional pressures and opportunities for development (de la Fuente and Martínez-Vicente, 2024). However, relatively few studies have examined in depth how university teachers interpret their emotional labor as part of their ongoing professional development and identity trajectories.

These gaps are particularly salient given calls to understand teacher well-being as a developmental and relational phenomenon. Cross-contextual research demonstrates that school and university climates that support basic psychological needs for autonomy, competence, and relatedness are associated with higher levels of well-being and more positive professional experiences (Harrison et al., 2025; Zhang et al., 2023). In contrast, integrative reviews of higher education stress highlight how persistent misalignment between institutional expectations and personal values can erode mental health and undermine career sustainability (de Sousa Pinho et al., 2023). Yet, we know relatively little about how university teachers themselves narrate the ways in which emotional demands, leadership practices, and digital or policy reforms shape not only their momentary feelings but also their evolving understandings of what it means to be a “good teacher,” how to sustain themselves emotionally, and whether to remain in academia. Addressing this gap requires qualitative, context-sensitive inquiry that takes seriously teachers’ own accounts of emotional labor, well-being, and professional growth.

Against this backdrop, the present study explores how university teachers experience and make sense of emotional labor in relation to their professional well-being and development. Drawing on an integrated framework that combines the JD-R model, emotional labor theory, and a professional development/identity perspective, we focus on three interrelated questions: how teachers describe the demands and resources that shape their emotional work; how they perceive the links between emotional labor strategies and their well-being; and how they weave emotional experiences into their longer-term professional trajectories. By employing in-depth interviews with university teachers in a specific higher education context, the study aims to complement existing quantitative research with rich, practice-near accounts, and to generate implications for institutional policies and development programs that seek to support more sustainable emotional lives for academic staff.

## Literature review

### Teacher well-being in higher education contexts

Contemporary research increasingly conceptualizes teacher well-being as a multidimensional construct that encompasses affective, cognitive, and relational aspects of professional functioning, rather than simply the absence of burnout or psychopathology. In the context of English language teaching, for example, ecological perspectives highlight how well-being emerges from dynamic interactions between teachers and their multi-layered environments, including institutional structures, professional communities, and transnational labor markets (Sadeghi and Pourbahram, 2024b). Professional learning and support structures have been shown to be central to this ecology: in an under-resourced region, a sustained professional development programmed improved English teachers’ well-being by strengthening their sense of competence, efficacy, and collegial connection, suggesting that well-being is tightly bound up with opportunities for growth and support (Wang and Chen, 2022).

Higher education is a particularly distinctive context in which teacher well-being is shaped not only by classroom demands but also by expectations related to research, service, and institutional change. Case-study work on university teacher preparation programmed shows that designing and implementing new courses, such as those focused on dyslexia, can both enrich teacher educators’ sense of purpose and increase their workload and emotional responsibilities, especially when they must mediate between policy expectations, institutional constraints, and student needs (Berryhill et al., 2025). Similarly, university-level reforms that emphasize sustainability and “teacher construction” as an instrument for higher education transformation point to increased symbolic and practical demands on academics as they are positioned as key agents of policy enactment (Ma et al., 2022). Studies in Mongolian universities further illustrate that teacher leadership and professional dispositions are central to building “sustainable and inclusive” institutions and are closely associated with student satisfaction, implying that teacher well-being cannot be disentangled from institutional development agendas (Ravdansuren et al., 2024).

At the same time, research on student well-being in higher education underscores the indirect but powerful significance of teachers and institutional climates. Work with adolescents and university students shows that psychological well-being is adversely affected by academic stress, problematic technology use, and school or university burnout, but these risks are mediated by learning environments and support structures (Kaya, 2024; Time et al., 2024). In Chinese universities, students’ psychological well-being has been linked to academic self-concept, perceived teacher support, and behavioral engagement, suggesting that teachers’ capacity to offer relational and instructional support is a critical resource for student flourishing (Zhang, 2024). Taken together, these strands of evidence position teacher well-being in higher education as a relational and ecological phenomenon, with implications not only for teachers’ own careers but also for students’ experiences and institutional quality.

Finally, teacher well-being is closely intertwined with resilience and career trajectories. Longitudinal qualitative work with beginning EFL teachers demonstrates that their resilience is co-constructed with professional development opportunities, mentoring, and the emotional challenges of early career teaching (Kim and Kim, 2024). Such studies suggest that well-being is not a static state but evolves over time, as teachers negotiate changing demands, supports, and identities. This developmental orientation is especially salient in higher education, where academic careers are often lengthy, non-linear, and marked by recurring cycles of adaptation to new roles, technologies, and policy regimes.

### Emotional labor in teaching

Emotional labor has become a key lens for understanding how teachers manage their feelings and emotional displays as part of their professional role. Building on Hochschild’s distinction between surface acting, deep acting, and genuine expression, recent work conceptualizes teaching as an intensively emotional practice that requires continual alignment, or strategic misalignment, between felt and displayed emotions. Multi-sited qualitative research with school teachers portrays emotional labor as “acting often and everywhere,” extending beyond classroom instruction to interactions with parents, administrators, and colleagues, and highlighting how emotional demands permeate the full spectrum of professional relationships (Levine Brown et al., 2023). In online spaces, teachers themselves have begun to resist reductive “well-being discourses,” with analyses of Reddit forums revealing how some educators critique institutional narratives that individualize responsibility for well-being and obscure structural sources of emotional strain (Karnovsky and Gobby, 2024).

Individual narratives and auto-ethnographic studies provide fine-grained insight into how emotional labor is lived and interpreted by teachers. A self-study of a volunteer teacher during the COVID-19 pandemic illustrates how identity negotiation and emotional labor intersect, as the teacher oscillates between enthusiasm, frustration, and vulnerability while attempting to live up to idealized images of care and professionalism in crisis conditions (Li, 2024). Similarly, language teachers engaged in online teaching have reported feeling compelled to project warmth, patience, and care “behind the screen,” even when they experience fatigue or anxiety, thereby intensifying their sense of emotional dissonance (Liu et al., 2023). These accounts underscore that emotional labor is not merely a set of strategies but is embedded in teachers’ self-understandings and their perceptions of what constitutes a “good teacher.”

At a more structural level, studies of school management cultures and early childhood settings show that organizational norms and leadership practices profoundly shape emotional labor patterns. In Mainland China, research on school management culture indicates that certain cultural configurations encourage high levels of surface acting and are associated with increased teacher burnout, whereas more supportive and participatory cultures are linked to more sustainable forms of emotional engagement (Tsang et al., 2021). Work with kindergarten teachers similarly finds that emotional labor strategies are influenced by contextual expectations and that they, in turn, are predictive of job satisfaction and emotional exhaustion (Yin et al., 2022). Within collaborative contexts, qualitative analyses of teacher collaboration reveal that emotional labor is not only an individual phenomenon but also a relational and reflective process: as teachers work together, they develop “emotional reflexivity” about how they manage and share emotions with peers (Song and Valentine, 2024). These findings collectively suggest that emotional labor is a central mechanism through which institutional demands and social norms are translated into teachers’ subjective experiences, with significant implications for well-being and professional sustainability.

### Teacher professional development and identity work

Teacher professional development and identity work constitute another crucial dimension of the literature that informs the present study. Rather than conceiving development as the linear acquisition of skills, recent research in higher education emphasizes identity construction, reflection, and negotiation of roles. Studies of university biology teachers, for instance, examine how academics craft “intelligible identities” in the figured worlds of higher education, revealing how they position themselves in relation to disciplinary norms, research expectations, and teaching ideals (Günter et al., 2022). In a similar vein, analyses of academic identities in contemporary universities argue that teachers and academics are seeking new ways of “being a university teacher” amid managerial reforms, performative accountability, and changing student demographics, highlighting identity work as a site of tension and creativity (Jiménez, 2024).

Professional development initiatives and teacher education structures play a key role in shaping these identities. Research on early childhood teacher education shows that university-based teacher educators construct practicum experiences through particular discourses, which in turn influence how pre-service teachers envisage their future roles and responsibilities (Kupila et al., 2023). Longitudinal work on pre-service teachers’ general pedagogical knowledge demonstrates that university learning opportunities and entry characteristics jointly shape knowledge development across multiple time points, underscoring that professional competence is formed through extended trajectories of learning rather than isolated interventions (Weyers et al., 2024). In the domain of literacy teaching, studies of professional development, curricular change, and coaching show that such initiatives alter teachers’ instructional practices and perceptions, with implications for their confidence, agency, and sense of professional growth (Brandt et al., 2025).

Higher education is also a site where professional development intersects with broader institutional agendas and technological change. Analyses of university strategies for sustainability indicate that “university teacher construction” is framed as a lever for achieving higher education sustainability, which places new symbolic and practical expectations on academics as drivers of change (Ma et al., 2022). Work on digital competence raises critical questions about whether digitally competent teachers are positioned as “agentic professionals in the digital age” or as “digitally compliant learning technicians,” suggesting that digitalization can both empower and constrain teacher identity (McGarr et al., 2025). Studies of teacher-leaders and professional dispositions in Mongolian universities similarly illustrate how institutional efforts to promote inclusion and student satisfaction hinge on particular constructions of what counts as a “professional” teacher (Ravdansuren et al., 2024). At the micro level, case studies of teacher-coaches in Japanese universities show how academics negotiate overlapping academic and coaching identities, navigating competing demands of performance, care, and scholarship (Mizushima et al., 2025). Finally, emerging work on systems that analyses university teachers’ classroom behaviors using hybrid deep learning techniques raises questions about surveillance, accountability, and the datafication of teaching, all of which are likely to reverberate through teachers’ identity work and emotional lives (Xie, 2025; Ruiz-Munzón et al., 2024).

Across these studies, a common thread is that teacher development in higher education is deeply entangled with identity, power, and institutional change. Professional growth is not merely about adopting new techniques or attending workshops; it involves ongoing negotiation of who teachers can be and how they can sustain themselves amid shifting demands, technologies, and expectations. This literature provides an important backdrop for examining how emotional labor and well-being are situated within broader professional trajectories.

### Integrated theoretical framework: JD-R, emotional labor, and teacher development

Synthesizing the strands reviewed above, the present study adopts an integrated framework that brings together the JD-R model, emotional labor theory, and perspectives on teacher professional development and identity work. From a JD-R perspective, university teachers’ work environments can be understood as configurations of job demands and job resources, complemented by personal resources such as resilience and emotion regulation skills. The literature on teacher well-being and professional learning indicates that these demands and resources jointly influence both teachers’ momentary affective states and their longer-term sense of professional sustainability.

Within this broader structure, emotional labor is conceptualized as a central process through which demands and resources are enacted in everyday practice. Teachers’ use of surface acting, deep acting, and more authentic emotional expression mediates the impact of institutional cultures, leadership practices, and professional learning opportunities on their experiences of well-being and ill-being, while also feeding back into their identity work and developmental trajectories. The identity-focused literature on higher education suggests that teachers continuously reinterpret their emotional experiences as part of wider narratives about what it means to be a “good” or “successful” academic, how to balance care and self-protection, and whether and how to remain in the profession.

**Figure 1:**
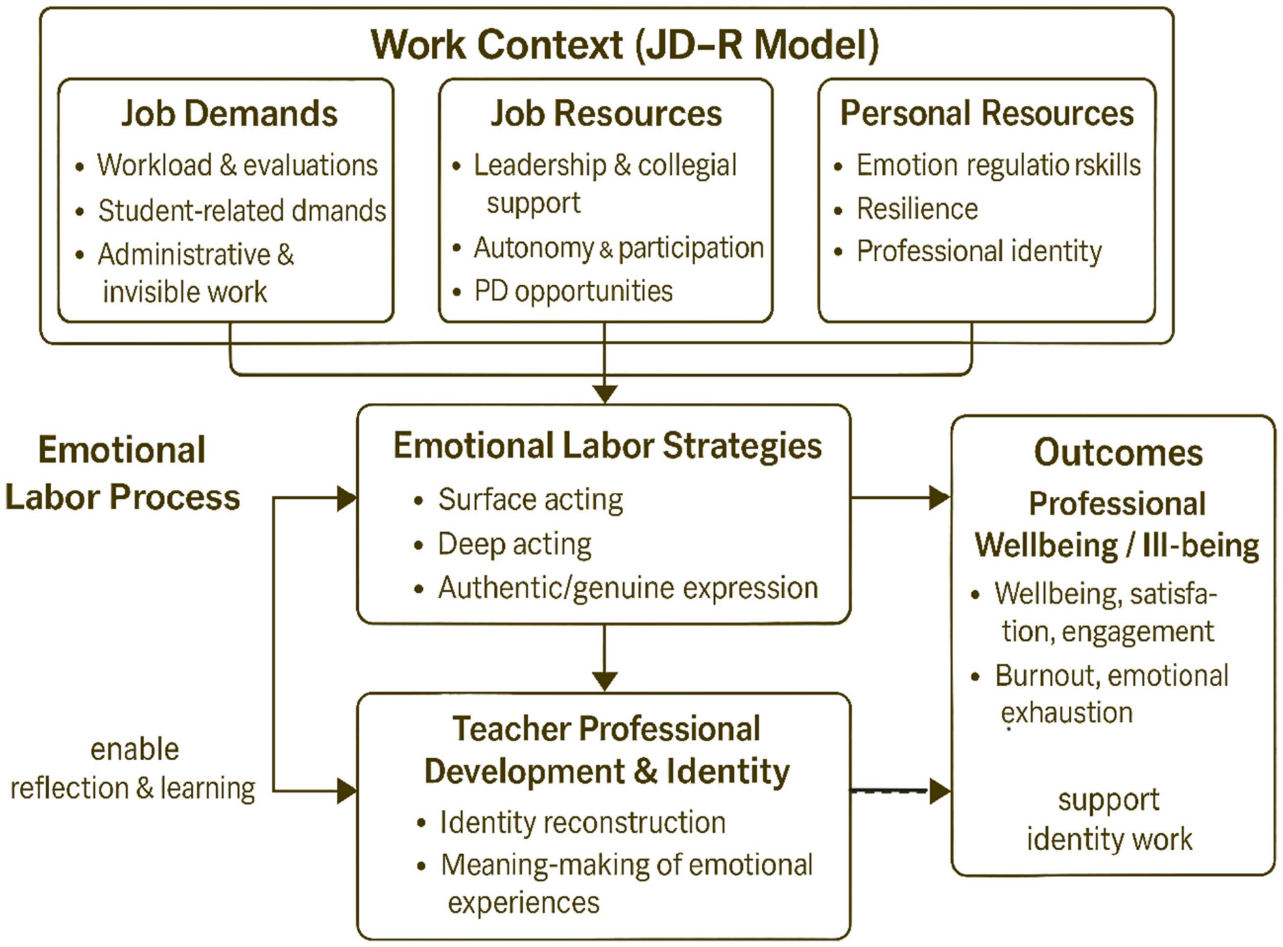
Conceptual framework of emotional labor, professional development and well-being in the JD-R context. Source: Developed by the authors.

This integrated conceptualization is depicted in Figure 1, which summarizes the proposed relationships between work context (job demands and resources, including personal resources), emotional labor strategies, and outcomes in terms of professional well-being and teacher development. In the empirical sections that follow, Figure 1 functions as a sensitizing framework rather than a rigid causal model: it guides the formulation of research questions and the analysis of interview data, while allowing teachers’ own accounts to refine, complicate, or challenge the assumed linkages.

## Methodology

### Research design

This study adopted an interpretivist qualitative research design to explore how university teachers experience and interpret emotional labor in relation to their professional well-being and development. Situated within a constructivist epistemological stance, the study assumes that emotional labor and well-being are not fixed psychological entities, but are socially and contextually constructed through teachers’ ongoing interactions with institutional structures, colleagues, and students. Accordingly, the aim was not to test predetermined hypotheses, but to generate in-depth, contextually grounded understandings of how teachers make sense of the emotional demands of academic work and how these meanings evolve across their professional trajectories.

Methodologically, the study can be characterized as a qualitative interview-based inquiry with a developmental and identity-oriented focus. It draws on elements of narrative inquiry insofar as participants were encouraged to recount critical incidents, turning points, and shifts in their understanding of care, professionalism, and self-protection over time. However, rather than producing full life-history narratives, the design centered on thematic patterns across participants’ accounts. Semi-structured, in-depth interviews were therefore employed to elicit rich descriptions of everyday emotional practices, perceived institutional expectations, and evolving coping strategies.

Data were analyzed using reflexive thematic analysis, following an iterative and interpretive process of coding, theme construction, and theoretical refinement. Reflexive thematic analysis was chosen because it aligns with an interpretivist paradigm and allows patterns of meaning to be developed inductively while remaining sensitized to the integrated theoretical framework that combines the JD-R model, emotional labor theory, and perspectives on teacher professional development and identity work. In this sense, the framework functioned as a sensitizing device rather than a deductive coding scheme, enabling the analysis to remain grounded in participants’ lived accounts while engaging in theoretically informed interpretation.

### Context and participants

The study was conducted in a higher education context characterized by increasing demands for teaching quality assurance, research productivity and student-centered service. The focal institution is a public, research-oriented university located in an urban setting, offering a broad range of undergraduate and postgraduate programmed. In recent years, the university has implemented reforms in areas such as digitalization of teaching, outcome-based education and performance evaluation, all of which shape the emotional texture of academic work and the expectations placed upon academic staff.

Participants were university teachers employed at this institution. A total of 10 teachers took part in the study. They were purposefully selected to represent variation in disciplinary background, career stage and gender, so that different constellations of demands and resources could be captured. Disciplines included, for example, the humanities and social sciences, education and selected fields in the sciences. At the time of interview, teaching experience ranged approximately from 3 years to more than 20 years, and participants held positions from lecturer to full professor. All participants were directly involved in undergraduate and/or postgraduate teaching and carried additional responsibilities such as student supervision, administrative roles or programmed coordination. To protect confidentiality, pseudonyms are used throughout the report and potentially identifying details, such as specific departments or modules, are omitted or aggregated.

### Sampling and recruitment

A purposive sampling strategy was employed to identify teachers who could provide information-rich accounts of emotional labor and well-being in university work. Initial participants were recruited through professional networks and targeted invitations circulated via institutional mailing lists. The basic inclusion criteria were that participants were currently employed as academic staff at the focal institution, had regular teaching duties and were willing to reflect on their emotional experiences in teaching and related academic work.

After the first round of interviews, further recruitment was guided by theoretical considerations derived from the emerging analysis and the integrated framework. In particular, attention was paid to including teachers who occupied high-contact teaching roles, those with substantial administrative loads and those who had recently navigated significant transitions such as promotion, assumption of leadership positions or shifts to online and hybrid forms of teaching. Recruitment continued until the research team judged that interviews were generating recurring patterns and conceptual redundancy in relation to the core themes of emotional demands and resources, emotional labor strategies, well-being experiences and professional development narratives. At that point, the dataset was considered sufficiently rich and diverse to address the research questions.

### Data collection

Data were collected through semi-structured, in-depth interviews conducted either face-to-face in a quiet office on campus or via secure video-conferencing platforms, depending on participants’ preferences and prevailing institutional conditions. Each interview lasted approximately 60–90 min and was conducted in a language shared by interviewer and participant. With participants’ consent, all interviews were audio-recorded.

The interview guide was informed by the integrated theoretical framework and organized around three broad areas, although the actual conversations were flexible and responsive to participants’ emphases. First, participants were invited to describe their current roles, typical teaching, research and administrative responsibilities, and the main sources of pressure and support they perceived in their work, including evaluation systems, leadership practices, collegial relationships, student expectations and ongoing institutional reforms. Second, they were asked to recount recent or memorable situations in which they felt they had to manage or “perform” emotions in teaching, supervision or collegial interactions, to elaborate on how they handled discrepancies between felt and displayed emotions and to reflect on the perceived short- and long-term effects of such emotional work on their energy, exhaustion, satisfaction and mental health. Third, they were encouraged to reflect on changes over time by discussing how their ways of dealing with emotions at work had evolved from earlier career stages to the present, which incidents or experiences reshaped their understanding of what it means to be a good teacher and what strategies they had developed to sustain themselves or reconsider particular roles.

The semi-structured format ensured that all key domains relevant to job demands and resources, emotional labor and professional development were covered, while leaving room for participants to introduce issues they considered important. Immediately after each interview, the researcher wrote brief field notes to record contextual details, non-verbal cues and initial analytic impressions. Audio recordings were subsequently transcribed verbatim and the transcripts were checked against the recordings to ensure accuracy.

### Data analysis

The interview data were analyzed using reflexive thematic analysis. The process began with intensive familiarization, during which the first author read and re-read all transcripts and listened again to selected audio excerpts to gain a holistic sense of each participant’s narrative. During this phase, initial notes were made about recurring emotions, tensions, metaphors and developmental storylines.

On this basis, initial coding was conducted line by line. Segments of text that related to work demands and resources, instances of emotional labor, experiences of well-being and ill-being and reflections on professional growth or identity were highlighted and assigned descriptive codes. Coding at this stage was deliberately open and close to participants’ own language so as not to impose theoretical categories prematurely.

As coding progressed, related codes were grouped and compared across cases, and candidate themes were developed that captured broader patterns of meaning, such as institutional emotional rules, the imperative to remain “professional” at all costs, the gradual learning of emotional boundaries or the reframing of emotional crises as opportunities for growth. The integrated framework of job demands and resources, emotional labor and teacher development functioned here as a sensitizing device, helping to keep analytic attention on relevant domains, but themes were not forced into a predetermined structure.

The candidate themes were then reviewed in relation to the entire dataset. This involved checking whether the themes cohered internally, whether they were sufficiently distinct from one another and whether important aspects of the data had been overlooked. During this phase, some themes were merged, some were divided into more focused subthemes and others were redefined or discarded. For each refined theme, the research team then worked out a clear thematic narrative, specifying the central organizing concept, its variations across participants and its connections to the research questions and theoretical concepts. Representative extracts were selected to illustrate each theme while preserving diversity of voices.

In the final stage of analysis, the thematic structure was related back to the conceptual model presented in Figure 1. The team examined how teachers’ accounts illustrated, extended or complicated the hypothesized relationships between job demands and resources, emotional labor strategies, professional well-being and teacher development. In this way, the analysis moved back and forth between inductive engagement with the data and theoretically informed interpretation. Qualitative data analysis software was used to assist in organizing codes, themes and memos, but analytic decisions remained interpretive and researcher-led.

### Trustworthiness and reflexivity

Several strategies were adopted to enhance the trustworthiness of the study. With regard to credibility, the researcher engaged with the data over an extended period, involving repeated readings and multiple cycles of coding and thematic refinement. Emerging interpretations were discussed within the research team in order to challenge assumptions, explore alternative readings and clarify the boundaries of themes. For a small subset of participants, preliminary thematic summaries were shared informally and their feedback was used to check whether the analysis resonated with their experiences and to identify possible misinterpretations.

Transferability was addressed by developing thick descriptions of the institutional context, participants’ roles and the nature of their work, particularly in the Findings section. These contextual details are intended to enable readers to assess the extent to which the findings may be applicable or insightful for other higher education settings. Dependability and confirmability were supported by maintaining an audit trail that documented key decisions during sampling, data collection, coding and theme development. Successive versions of the codebook, thematic maps and analytic memos were retained so that the evolution of the analysis could be traced.

Reflexivity was treated as integral rather than optional. The lead researcher’s own experience as a university teacher and researcher was explicitly acknowledged, including personal investments in issues such as workload, evaluation and caring for students. Throughout the project, reflexive memos were written to examine how these positionalities might shape the questions asked, the aspects of participants’ account that attracted attention and the interpretations advanced. Instead of aspiring to full neutrality, the research team sought to make these influences visible and to use them as a resource for deeper and more critical engagement with the data.

### Ethical considerations

The study complied with institutional and disciplinary ethical standards for research involving human participants. Prior to data collection, ethical approval was obtained from the relevant university research ethics committee. Potential participants received an information sheet that outlined the purpose of the study, what participation would involve, the voluntary nature of their involvement and the measures that would be taken to maintain confidentiality and anonymity. Written informed consent was obtained from all participants before interviews began.

During the interviews, participants were reminded that they could decline to answer any question and could withdraw from the study at any point without any negative consequences. Particular care was taken when discussing potentially sensitive topics such as mental health, burnout or conflicts with students and colleagues; the interviewer monitored signs of discomfort and offered to pause or redirect the conversation when necessary. To protect anonymity, pseudonyms are used in all written outputs and identifying details have been removed or altered. Audio files and transcripts were stored on password-protected drives with access restricted to the research team, and data management followed institutional policies regarding secure storage and eventual disposal of research data.

## Results

### Theme 1: Institutional emotional rules and hidden pressures

Across interviews, participants described a pervasive set of implicit “emotional rules” that shaped how they felt they ought to present themselves in the university. Several spoke of a tacit expectation that a competent academic is calm, composed and endlessly resilient. T7 summarized this as “an unwritten policy that you never lose your temper, never show panic, never say ‘I cannot cope’,” and T3 added that “the culture here is that you carry everything with a smile; if you show cracks, people start questioning your professionalism.” These accounts suggest that emotional restraint and positivity are treated as part of academic competence, even though they are rarely acknowledged explicitly (see Table 1).

**Table 1:** Interview themes.

Theme	Subtheme	Brief description
Theme 1. Institutional emotional rules and hidden pressures	“Always positive, always available”	Perceived expectations to be endlessly patient, enthusiastic, and emotionally available to students, colleagues, and administrators
	Metrics, evaluations, and invisible emotional work	How student evaluations, performance indicators, and accountability regimes generate hidden emotional demands
	Blurred boundaries between work and private life	The spillover of emotional demands beyond formal working hours and spaces, and the difficulty of “switching off”
Theme 2. Navigating emotional labor: strategies and costs	“Acting professional” through surface acting	Use of surface acting to suppress frustration, fatigue, or anxiety and display institutionally desired emotions
	Deep acting and compassion as relational work	Efforts to genuinely reframe situations and cultivate empathic concern in order to feel and display more congruent emotions
	Setting emotional boundaries and selective authenticity	Developing ways to express emotions more honestly and to protect personal limits, without abandoning professional responsibilities
Theme 3. Emotional labor as a site of professional learning and development	Emotional crises as turning points	Moments of exhaustion, conflict, or breakdown that prompt rethinking of teaching, care, and self-protection
	Reframing care and responsibility across career stages	Shifts in how teachers define “being a good teacher” and what they owe to students and institutions over time
	Building sustainable practices and collective support	Gradual construction of more sustainable emotional practices, including peer support, mentoring, and renegotiation of roles

A recurring motif was the requirement to be “always positive, always available,” especially in relations with students. T2 explained that “students expect you to be friendly, supportive, answering messages at any time… if you are not ‘nice’ enough, they write it in the evaluation,” while T5 felt that “the emotional demand is not just to teach well but to be the kind of teacher who is endlessly patient and understanding.” Several teachers described replying to messages late at night because they feared being perceived as uncaring. As T1 put it, “you feel guilty if you ignore a message, even at midnight, because you know there is a scoreboard somewhere, and one unhappy student can affect your record.” In this sense, availability was not only a personal preference but an affective obligation tied to institutional surveillance.

Teachers also stressed that metrics and evaluations intensified emotional demands in subtle ways. T8 noted that “student evaluations are like an emotional exam every semester; you are waiting to see if they like your ‘performance’,” while T1 described how “one sarcastic comment in the feedback can haunt you for days; you replay the whole semester thinking what you did wrong.” Beyond teaching evaluations, publication targets and grant expectations created a continuous background pressure that seeped into classroom interactions. T6 reflected that “when you are under pressure to publish, you start to see every question from students as a potential distraction, and then you have to suppress that irritation because, officially, teaching should be your passion.” The emotional work of aligning oneself with these competing expectations remained largely invisible in formal workload models.

The blurring of boundaries between work and private life further amplified these pressures. Many participants described feeling as if they were “never really off duty.” T4 commented that “my phone is basically an extension of my office; students WhatsApp, admin emails, all mixed with family messages,” and T9 remarked that “even when I’m putting my child to bed, in my head I’m composing replies to students … it’s like a second layer of thinking that never stops.” Attempts to switch off were often accompanied by guilt or anxiety about missing something important. T2 admitted, “Sometimes I switch my phone to airplane mode, and then I feel like a bad teacher, like I am abandoning them.” Over time, this low-level but continuous vigilance contributed to feelings of depletion and detachment.

Despite sharing these pressures, some participants also described moments of subtle resistance to institutional emotional rules. T6, a senior academic, recounted deliberately refusing to respond immediately to late-night student complaints: “I decided that my sleep is more important than a perfect image. I answer during working hours and I tell them this is part of being an adult.” T10 similarly observed that “if we all keep playing the superhero, the system will never change; sometimes you have to say, ‘No, this is not reasonable’.” However, even these acts of boundary-setting were narrated cautiously, with teachers weighing the risks to their reputation and promotion prospects. Overall, Theme 1 highlights how institutional norms, evaluation regimes and digital connectivity together produce a dense web of hidden emotional pressures that frame what it means to be a “good” university teacher.

### Theme 2: Navigating emotional labor—strategies and costs

In response to these demands, participants described a repertoire of emotional labor strategies that they mobilized in everyday work. A first layer of this repertoire involved what many called “acting professional,” which they primarily associated with surface acting. T1 described her routine before class as “putting on the professional face,” explaining that “even if I had a terrible morning, I take a deep breath at the door, fix my expression and tell myself, ‘Now you are the competent lecturer; your feelings can wait’.” T3 echoed this sentiment, saying that “students do not come to see your bad mood; they come to see someone who knows what they are doing,” which often meant hiding confusion or irritation. For early-career staff in particular, surface acting was framed as a survival strategy in a context where reputation was still fragile.

The costs of sustained surface acting, however, were repeatedly emphasized. T10 recounted a semester where she felt she was “performing happiness every day” while dealing with family illness: “I smiled, I joked, I was the energetic teacher… and then I went home and could not speak to anyone; I was completely empty.” T3 described a similar pattern: “after a day of pretending I’m calm and okay, I feel like I have no emotions left for my own family.” Some participants noted that long-term reliance on surface acting led to a sense of disconnection from their genuine emotions. As T5 put it, “after a while you do not even know if you are really enjoying the class or just performing enjoyment because that’s what is expected.” These accounts point to emotional numbing and fragmentation as potential consequences of habitual surface acting.

Alongside surface acting, many teachers reported efforts to engage in deeper forms of emotional work. T7 explained that “if I only pretend to care, students feel it; so I try to understand what is behind their behavior, to really shift my perspective before I respond.” In describing a student who repeatedly missed deadlines, T5 recounted: “I was very angry at first, but then I asked myself, ‘What else could be going on?’ After talking to him, I realized there were family issues. That changed how I felt; I moved from anger to concern.” Participants characterized this kind of reframing and empathic engagement as emotionally demanding but also more satisfying than mere suppression. T2 noted, “when I manage to genuinely feel more patient, I sleep better afterwards than when I just bite my tongue.” Nevertheless, deep acting was described as a finite resource: “you cannot do deep empathy with everyone every day,” T9 observed, “otherwise you drown in other people’s stories.”

A further strategy involved setting clearer emotional boundaries and engaging in what participants termed “selective authenticity.” T2 described learning to “be honest without dumping everything,” explaining that “now if I’m exhausted, I might say to the class, ‘I’m a bit-tired today, so let us work together to make this session productive.’ It’s authentic but still professional.” T9 spoke of gradually allowing herself to express mild frustration when administrative demands were excessive: “I used to say yes to everything with a smile. Now I sometimes say, ‘I’m already overloaded; I cannot take this on right now.’ It is scary, but it also feels like I am respecting myself.” For several participants, this selective authenticity was closely associated with a more grounded sense of professional identity, in which being a good teacher did not mean endless self-sacrifice.

Teachers were acutely aware of the trade-offs involved in these strategies. T4, a mid-career academic, described oscillating between surface acting and authenticity: “if I show too much of my real emotions, I worry that students will think I’m unprofessional or weak; if I show nothing, I feel like a robot.” T6 framed the issue in terms of institutional constraints: “we can only be authentic within certain boundaries set by the system; you can say you are tired, but you cannot say you are angry at the policy that created this situation.” These reflections underline that emotional labor was not simply a matter of individual choice but was negotiated within structural limits. At the same time, participants suggested that over time they became more deliberate and strategic in how they combined surface acting, deep acting and authenticity in order to protect their well-being while still meeting professional expectations.

### Theme 3: Emotional labor as a site of professional learning and development

Beyond describing pressures and strategies, participants also reflected on how emotional labor had shaped their longer-term professional development and identity. Many framed particularly intense periods of emotional strain as turning points that forced them to rethink their approach to teaching and self-care. T8 recalled a year when, after a series of negative student evaluations and missed research deadlines, he experienced what he called “a mini burnout”: “I was crying in my office, thinking, ‘Maybe I am not cut out for this job.’ That crisis pushed me to ask, ‘What kind of teacher do I want to be, and how can I do this without destroying myself?’.” Similarly, T1 described a conflict with a group of students as “the moment I realized that trying to please everyone is impossible and unhealthy.” These crises were painful but were retrospectively interpreted as catalysts for more sustainable ways of working.

Participants also described gradual shifts in how they understood care and responsibility across career stages. Early in their careers, several felt that being a good teacher meant unconditional availability and emotional investment. T3 stated that “in my first years, I believed I had to carry every student’s problem on my shoulders; otherwise, I was failing them.” With time, however, some began to differentiate between “caring for” and “carrying” students. T5 explained: “now I still care deeply, but I don’t assume that I must solve everything. I see my role more as guiding and connecting them to other resources.” This reframing reduced feelings of guilt when boundaries had to be set and was associated with a stronger sense of professional legitimacy.

Differences between career stages were particularly evident in how teachers narrated their relationship to institutional expectations. T2, an early-career lecturer, spoke of feeling “constantly under probation,” which made it difficult to resist emotional demands: “when you are new, you say yes to everything, you absorb everything; you don’t want to be labelled as ‘difficult’.” In contrast, more experienced teachers such as T7 felt better able to negotiate: “after 15 years, I know my value and my limits. I am still committed, but I am no longer willing to sacrifice my health for every institutional project.” T7 linked this shift directly to accumulated emotional experience, saying that “the emotional scars from earlier years taught me where my boundaries are.” These narratives suggest that emotional labor is intertwined with professional maturation and changing power positions within the institution.

Collective practices and relationships emerged as another important dimension of learning to manage emotional labor. T4 described an informal lunchtime group with colleagues as “our therapy session,” where they shared stories of difficult classes and laughed about bureaucratic absurdities: “it doesn’t change the system, but it changes how heavy it feels.” T9 mentioned the role of mentoring: “my senior mentor told me, ‘You are not a bad teacher if you say no.’ That sentence stayed with me and gave me permission to protect myself.” T10 emphasized the significance of talking openly about emotions in departmental meetings: “when senior people admit they also feel overwhelmed sometimes, it normalizes our feelings and makes it easier to seek help.” Such collective spaces enabled participants to reframe individual struggles as shared structural issues and to experiment with alternative emotional practices.

Finally, several teachers explicitly linked their evolving emotional practices to the development of a more coherent professional identity. T6 reflected that “for a long time, my identity was split: the official ‘strong’ academic and the exhausted person at home. Slowly, I am integrating these, accepting that vulnerability is part of being a teacher.” T5 similarly concluded that “learning how to deal with emotions is not extra to my development; it is at the heart of becoming the kind of teacher I want to be.” In this sense, emotional labor was not only a source of strain but also a site where teachers actively worked on themselves, re-negotiating what it means to be a university teacher and how to sustain a career in higher education. These accounts resonate with the integrated theoretical framework by showing how job demands and resources, emotional labor strategies and well-being co-evolve over time within teachers’ developmental trajectories.

## Discussion

The present study sought to interpret how university teachers experience and negotiate emotional labor within contemporary higher education contexts. While the Results section outlined the thematic structure emerging from participants’ narratives, the present discussion situates those findings within broader theoretical, institutional and cultural debates.

Within a JD-R perspective, the data indicate that emotional labor is deeply embedded in institutionalized performance cultures characterized by intensified evaluation regimes, digital connectivity and normative expectations of continuous availability. The recurrent expectation to remain “always positive” and “always available” reflects not merely interpersonal norms but structurally embedded professional scripts. These scripts align care, responsiveness and productivity with institutional legitimacy. Emotional regulation thus becomes a tacit performance requirement, operating as an invisible extension of workload rather than as a formally recognized dimension of academic labor.

At the same time, participants’ accounts complicate any unidirectional interpretation of emotional labor as either purely detrimental or inherently developmental. While some teachers described moving toward deep acting, boundary-setting and selective authenticity, this was neither universal nor linear. Several accounts revealed ambivalence, emotional withdrawal and persistent strain. A minority of participants described narrowing student interaction, distancing themselves from institutional expectations or contemplating exit from academic roles when emotional demands became overwhelming. These negative and contradictory cases underscore that emotional labor may simultaneously function as a site of identity reconstruction and as a mechanism of erosion. Recognizing these tensions is critical to avoiding the normalization or romanticization of emotional labor as an inevitable pathway to professional growth.

The findings further suggest that institutional and cultural contexts require more explicit theoretical consideration. The university examined in this study operates within a broader system shaped by audit cultures, competitive funding logics and globalized performance metrics. Such macro-level dynamics contribute to what may be conceptualized as an “availability culture,” in which emotional responsiveness becomes conflated with professional competence. Emotional demands are therefore co-produced by policy climates, governance structures and relational expectations across students, colleagues and administrators. Teacher well-being in higher education should thus be conceptualized ecologically, as situated within interacting institutional and cultural systems rather than as an individual psychological attribute.

In relation to wider psychological well-being literature, parallels can be observed with other caregiving and relational professions where coping strategies mediate the impact of chronic demands (Matera et al., 2024; Redican et al., 2024; Rutter et al., 2024). Similarly, research on identity and connectedness in emotionally intensive professional roles (Shaughnessy et al., 2023) reinforces the importance of relational and communal resources. Evidence from structured psychosocial interventions in other high-demand settings further suggests that emotional awareness and reflective capacity can buffer strain when embedded within supportive systems (Tabib et al., 2024; Telles et al., 2024). However, translating such insights to higher education requires caution. Interventions that focus solely on individual resilience risk obscuring the structural sources of emotional strain. Institutional responsibility for workload regulation, evaluation practices and communication norms remains central.

Several limitations warrant careful acknowledgment. The small sample (*n* = 10) drawn from a single institution limits transferability. The study illuminates’ processes and meanings rather than prevalence or generalizable patterns. Institutional culture, disciplinary context and national higher education policy frameworks may significantly shape emotional labor dynamics. Comparative and cross-cultural research is necessary to examine how structural configurations moderate these processes. Furthermore, although efforts were made to identify divergent and contradictory cases, additional exploration of marginalized or dissenting experiences would deepen understanding of how emotional labor intersects with career stage, gender, employment precarity or disciplinary positioning.

Reflexively, the research team recognizes that our interpretations are shaped by our own positioning within academic systems. Although interviews were conducted by one author, analytic development was collaborative, involving iterative discussion among all authors to challenge assumptions and refine thematic interpretations. Nonetheless, the analysis remains a situated account rather than an objective representation of reality, consistent with an interpretivist stance.

Overall, the findings suggest that emotional labor in higher education should neither be pathologized nor normalized. It constitutes a dynamic field in which institutional demands, personal resources and identity processes intersect. Sustainable professional well-being requires structural as well as individual attention.

## Conclusion

This study explored how university teachers negotiate emotional labor within increasingly performance-driven and digitally intensified higher education environments. The findings show that implicit norms of positivity, availability and relational attentiveness function as powerful yet often unacknowledged job demands, shaping teachers’ daily practices and self-presentations. Teachers respond through shifting combinations of surface acting, deep acting and selective authenticity, with sustained surface acting associated with depletion, and more reflective boundary-setting linked to greater professional coherence. Emotional labor thus emerges as a contested arena in which strain and professional learning coexist, but its developmental potential is neither automatic nor evenly distributed. By integrating the JD-R model with emotional labor theory and perspectives on professional identity, the study offers a contextually grounded account of how work conditions, emotional practices and well-being co-evolve in higher education. At the same time, the small, single-institution qualitative design limits transferability and underscores the need for comparative, longitudinal and mixed-methods research to examine how institutional structures, policy environments and targeted interventions shape emotional labor trajectories across contexts and career stages. Moving beyond individualized narratives of resilience, the findings highlight the importance of institutional responsibility in regulating structural demands while creating collective spaces in which emotional experiences can be critically reflected upon and reinterpreted.

## Data Availability

The datasets generated and analyzed for this study consist of interview transcripts that contain potentially identifying information. In order to protect participants’ confidentiality, full transcripts are not publicly available. De-identified excerpts or analytic materials can be provided by the corresponding author upon reasonable request and in accordance with institutional ethical regulations.
